# Epigenome-Wide Analyses Identify Two Novel Associations With Recurrent Stroke in the Vitamin Intervention for Stroke Prevention Clinical Trial

**DOI:** 10.3389/fgene.2018.00358

**Published:** 2018-09-06

**Authors:** Nicole M. Davis Armstrong, Wei-Min Chen, Michael S. Brewer, Stephen R. Williams, Michèle M. Sale, Bradford B. Worrall, Keith L. Keene

**Affiliations:** ^1^Department of Biology, East Carolina University, Greenville, NC, United States; ^2^Center for Public Health Genomics, University of Virginia, Charlottesville, VA, United States; ^3^Department of Public Health Sciences, University of Virginia, Charlottesville, VA, United States; ^4^Department of Neurology, University of Virginia, Charlottesville, VA, United States; ^5^Center for Health Disparities, East Carolina University, Greenville, NC, United States

**Keywords:** DNA methylation, recurrent stroke, VISP, association, folate one carbon metabolism, homocysteine, epigenome, epigenetics

## Abstract

DNA methylation, a well-characterized epigenetic modification that is influenced by both environment and genetic variation, has previously been implicated in a number of complex diseases, including cardiovascular disease and stroke. The goal of this study was to evaluate epigenome-wide associations with recurrent stroke and the folate one-carbon metabolism-related trait, plasma homocysteine (hcy). Differential methylation analyses were performed on 473,864 autosomal CpG loci, using Illumina HumanMethylation 450K array data in 180 ischemic stroke cases from the Vitamin Intervention for Stroke Prevention (VISP) clinical trial. Linear regression was used to assess associations between number of strokes prior to VISP enrollment and measures of hcy with degree of methylation (β-values), while logistic regression was used to evaluate recurrent stroke status and incident recurrent stroke associations. All regression analyses were stratified by race. Two differentially methylated CpG sites exceeded epigenome-wide significance (*p* ≤ 1.055 × 10^−7^) for prior number of strokes (PNS) in European Americans. The top locus, cg22812874, was located in the ankyrin repeat and SOCS box containing 10 gene (*ASB10*; *p* = 3.4 × 10^−9^; β = −0.0308; 95% CI = −0.040, −0.002). Methylation locus cg00340919, located in an intron of the tetratricopeptide repeat domain 37 gene, was also statistically significant (*TTC37*; *p* = 8.74 × 10^−8^; β = −0.0517; 95% CI = −0.069, −0.034). An additional 138 CpG sites met our threshold for suggestive significance (*p* ≤ 5 × 10^−5^). We evaluated DNA methylation associated with recurrent stroke and hcy phenotypes across the epigenome. Hypermethylation at two CpG sites located in *ASB10* and *TTC37* was associated with fewer strokes prior to VISP enrollment. Our findings present a foundation for additional epigenome-wide studies, as well as mechanistic studies into epigenetic marks that influence recurrent stroke risk.

## Introduction

Epigenetics refers to chemical modifications in DNA that influence and regulate gene expression without altering the sequence. In mammalian cells, DNA methylation occurs with a covalent addition of a methyl group to a cytosine primarily located at a CpG dinucleotide (Li et al., 1992; Robertson, 2011) and is influenced by both environmental and genetic factors (Jirtle and Skinner, 2007; Mathers et al., 2010). Aberrant DNA methylation patterns have been implicated in a number of diseases (Robertson, 2005). The FOCM pathway plays a crucial role in DNA methylation through the conversion of methionine to homocysteine (hcy) with the aid of B vitamins (Fox and Stover, 2008). Elevated levels of plasma hcy have been associated with an increased risk of stroke (Boushey et al., 1995; Wald et al., 2005; Shi et al., 2015).

Annually, stroke contributes to 6.5 million deaths worldwide and is the fifth leading cause of death in the United States (Benjamin et al., 2017). There are roughly 795,000 reported cases of stroke in the United States each year and nearly a quarter are recurrent attacks, which are often more disabling and fatal compared to an initial stroke. Approximately 85% of strokes are ischemic attacks; a complex disorder with both genetic and environmental contributions (Benjamin et al., 2017). After adjusting for the major risk factors contributing to ischemic stroke, an estimated 69% of the population-attributable risk is not accounted for (Jamrozik et al., 1994; Benjamin et al., 2017), suggesting genetic and epigenetic factors may account for a substantial proportion of stroke risk (Jamrozik et al., 1994; Qureshi and Mehler, 2010).

A more mechanistic understanding of the relationship between the FOCM pathway, hcy, DNA methylation, and vascular disease could help identify prevention strategies for stroke. This study performed epigenome-wide associations with recurrent stroke and FOCM-related phenotypes, using methylation data of 180 participants from the VISP clinical trial. Our findings implicate two novel methylation loci associated with the number of strokes suffered prior to trial enrollment.

## Materials and Methods

### Ethics Approval

The institutional review boards (IRBs) of Wake Forest School of Medicine and the University of North Carolina at Chapel Hill School of Medicine approved the VISP clinical trial study protocol. The local IRB for each of the individual recruiting sites approved the VISP protocol and all participants provided written, informed consent. The genetic and epigenetic components of the VISP study were approved by the IRBs at the University of Virginia School of Medicine and East Carolina University.

### Subjects

Vitamin Intervention for Stroke Prevention was a multicenter, double-blind, randomized, and controlled clinical trial designed to determine whether folic acid, pyridoxine (B_6_), and cyanocobalamin (B_12_) supplementation, in addition to best medical management, prevented recurrent cerebral infarction and reduced the risk for myocardial infarction or fatal coronary heart disease in participants aged 35 years or older. VISP participants with baseline hcy levels above the 25th percentile were enrolled within 120 days of suffering a NDCI characterized by the sudden onset of a neurologic deficit persisting for at least 24 h and not due to a cardiac embolism (Toole, 2002). Patients (*n* = 3,860) were randomly assigned to receive daily doses of either the high-dose (*n* = 1,827; 25 mg B_6_, 0.4 mg B_12_, and 2.5 mg folic acid) or the low-dose formulation (*n* = 1,853; 200 μg B_6_, 6 μg B_12_, and 20 μg folic acid). A subset of 2,100 VISP participants provided consent for inclusion in genetic studies, of which methylation data was generated for 204 participants. Although all VISP participants had elevated baseline hcy levels, the 204 participants from the methylation subset were selected at the extremes (highest and lowest) for hcy levels. After filtering, quality control, and normalization, methylation data from 180 individuals were used in subsequent analyses (104 of European descent and 76 of African descent).

### FOCM Metabolite Measurements

Baseline fasting and 2 h post methionine-load hcy levels were determined at randomization for all VISP participants. Each participant fasted overnight, gave a whole blood sample for baseline measurements and was subsequently given methionine (0.1 g/kg body weight) as a crystalline powder dissolved in unsweetened juice. Two hours after consumption, another blood sample was taken to determine post-methionine hcy level (Toole et al., 2004; Pettigrew et al., 2008). The absolute difference between the baseline and post-methionine load hcy (DeltaPost) was calculated and provides a measurement of an individual’s ability to convert methionine into homocysteine.

### Methylation Data Generation

DNA methylation data was generated using the Illumina Infinium HumanMethylation450K BeadChip microarray (Illumina, Inc., San Diego, CA, United States) per the manufacturer’s protocol. Denaturation and sodium bisulfite conversion of genomic DNA was performed using Zymo EZ DNA Methylation Kits (Zymo Research Corporation, Irvine, CA, United States). The BCD was eluted from the Zymo-Spin column, amplified, enzymatically fragmented, precipitated, and resuspended. This solution was hybridized to the BeadChip for 22 h at 48°C and BeadChips were subsequently washed to remove unhybridized DNA samples. Single base extensions of hybridized primers labeled with biotin underwent staining. The BeadChips were washed, coated, and imaged on an iScan (Illumina, Inc., San Diego, CA, United States) (Maksimovic et al., 2012).

Imaged data was viewed using GenomeStudio (Illumina, Inc., San Diego, CA, United States). The degree of methylation, or β-value, was calculated as the ratio of methylated allele intensity divided by the sum of the intensities of methylated and unmethylated loci for 485,512 CpG sites. Quality control and quantile normalization were performed using the Bioconductor Minfi and watermelon packages, respectively (Touleimat and Tost, 2012; Pidsley et al., 2013; Aryee et al., 2014). Removal of methylation loci located on sex chromosomes resulted in 473,864 autosomal methylation sites that were included in subsequent analyses. Raw DNA methylation data, linked to genome-wide association SNP data, will be available at the NCBI database of Genotypes and Phenotypes (dbGaP Study Accession: phs000343.v3.p1).

### Statistical Analyses

Principal components analysis was performed on SNP data acquired from VISP genetic studies using KING software (Manichaikul et al., 2010). The top four and ten principal components were used as covariates in regression analyses to account for population substructure and admixture in EA and AA, respectively.

Cellular heterogeneity due to variation in cell population proportions in whole blood (Reinius et al., 2012) was controlled for using cell proportion estimates based upon the Houseman algorithm (Houseman et al., 2012). Estimated cell counts were generated for B-lymphocytes, CD4+ and CD8+ T-lymphocytes, natural killer cells, granulocytes, and monocytes using the “estimateCellCounts()” function in the R package minfi (Aryee et al., 2014; Jaffe and Irizarry, 2014) and were used as covariates in regression models, as described below.

To identify differentially methylated CpG sites associated with measures of baseline plasma hcy (hcy), post-methionine load hcy (POST), and the absolute difference (DeltaPost), a multiple linear regression stratified by race and adjusting for age, sex, principal components, batch, and estimated cell proportions was performed in R (version 3.3.1).

To identify CpG sites associated with recurrent stroke (experiencing more than one stroke in one’s lifetime, “Recurrent Ever”; incident stroke during the VISP trial, “VISP Recurrent”), a logistic regression stratified by race and adjusting for age, sex, principal components, batch, estimated cell proportions, and trial arm (high- or low-dose supplementation) was performed. Differentially methylated CpG sites associated with the number of strokes prior to enrollment (PNS) were analyzed using a linear regression model adjusted for age, sex, principal components, batch, and estimated cell proportions. Two individuals (one AA and one EA) were excluded in this model due to dependent variable outliers.

Significant associations were corrected for multiple testing using a Bonferroni-adjusted epigenome-wide threshold of 1.055 × 10^−7^. The beta coefficients and 95% confidence intervals (CIs) for 473,864 methylation loci were calculated in R (**Supplementary Tables S1**, **S2**). Manhattan plots were generated using qqman (Turner, 2018) and lambda values were estimated using GenABEL (Aulchenko et al., 2007).

Statistical power was calculated for both AA and EA subpopulations at an effect size of 0.15, significance level of α = 0.05, and degrees of freedom corresponding to race subpopulation size using the pwr package (Cohen, 1988; Champely, 2017). The AA subpopulation resulted in limited power of 0.327, while the EA subpopulation had power of 0.643.

### Gene Ontology Term Enrichment

Gene ontology (GO) term enrichment was performed using the GOrilla (Gene Ontology enRIchment anaLysis and visuaLizAtion) tool (Eden et al., 2007, 2009) and the missMethyl package in R (Phipson et al., 2016). The top 86 unique genes associated with differentially methylated CpG sites were ranked and compared to a background list of 15,057 terms for biological processes, and 4,518 terms for molecular function in the GOrilla program (*p* ≤ 3.321 × 10^−6^ and *p* ≤ 1.107 × 10^−5^, respectively). The same subset of unique genes was used in missMethyl across 20,732 terms (*p* ≤ 2.411 × 10^−6^).

## Results

**FIGURE 1:**
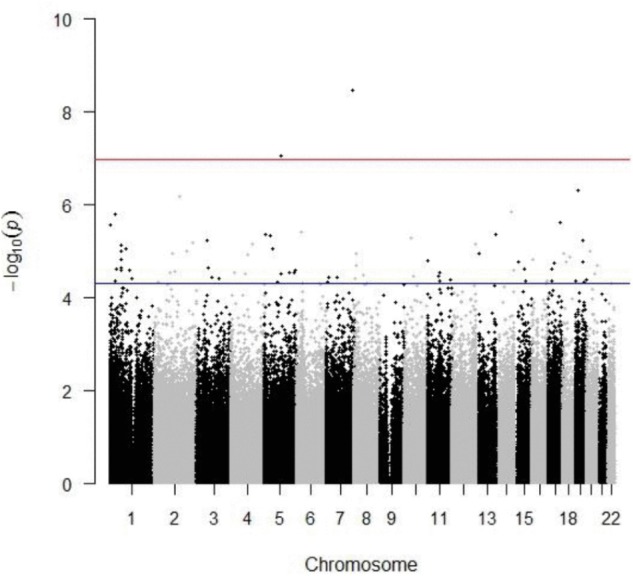
Manhattan plot depicting results from linear regression of previous number of strokes in European American population. The red line indicates a Bonferroni-adjusted threshold of 1.055 × 10^−7^; blue line indicates suggestive significance threshold of 5.0 × 10^−7^. Inflation value, = 1.31.

DNA methylation data was generated for a subset of 180 individuals from the VISP trial (AA *n* = 76; EA *n* = 104; **Table 1**), using the Illumina 450K array. Linear and logistic regression for recurrent stroke phenotypes (PNS, VISP Recurrent, Recurrent Ever) identified two statistically significant (*p* ≤ 1.055 × 10^−7^) CpG sites in EA (**Table 2** and **Figure 1**). The most significant association was observed at cg22812874 (*p* = 3.40 × 10^−9^, β = −0.0308; 95% CI = −0.040, −0.002), located in exon 6 of the ankyrin repeat and SOCs box containing 10 gene, *ASB10*, on chromosome 7. This locus is also found in a TFBS for the CCCTC-binding factor, CTCF. Methylation locus cg00340919, located within intron 4 of the tetratricopeptide repeat domain 37 gene, *TTC37*, on chromosome 5, also exceeded epigenome-wide significance (*p* = 8.74 × 10^−8^, β = −0.0517; 95% CI = −0.069, −0.034). A subset of 82 CpG sites reached an *a priori* suggestive significance threshold of *p* ≤ 5 × 10^−5^ (**Supplementary Figures S7**–**S11** and **Supplementary Table S1**).

**Table 1:** Demographic summary statistics.

	AA	EA	Total	*p*-Value
Number of individuals	76	104	180	
**Age (years)**				
Mean ± SD	62.16 ± 10.17	68.63 ± 11.01	65.90 ± 11.11	8.42 × 10^−5∗^
Range	42–84	40–87	40–87	
Sex	M: 46 | F: 30	M: 57 | F: 47	M: 103 | F: 77	0.444†
**Number of strokes prior to VISP**				
Mean ± SD	0.8684 ± 2.39	0.4712 ± 1.11	0.6389 ± 1.78	0.139^∗^
Range	0–4	0–3	0–4	
% VISP Recurrence^§^ (*n*)	36.84% (28)	30.77% (32)	33.33% (60)	0.393†
% Recurrence Ever^¶^ (*n*)	57.89% (44)	45.19% (47)	50.56% (91)	0.092†
**Measured baseline homocysteine**				
Mean ± SD	15.04 ± 6.09	16.28 ± 11.35	15.76 ± 9.49	0.331^∗^
**Measured post-methionine load test homocysteine**				
Mean ± SD	29.57 ± 12.21	33.62 ± 15.38	31.92 ± 14.24	0.107^∗^

*^∗^p-value calculated by t-test; ^†^p-value calculated by chi-square test; ^§^VISP Recurrence: recurrent stroke during the VISP clinical trial; ^¶^Recurrence Ever: individual suffered stroke prior to enrollment (PNS) or during VISP clinical trial.*

**Table 2:** Statistically significant (*p* ≤ 1.055 × 10^−7^) differentially methylated sites associated with the previous number of strokes in European Americans.

Locus	CHR	BP^∗^	Beta coefficient (95% CI)	SE	*t*-stat	*p*-Value	Gene
cg22812874	7	150872892	−0.0308 (−0.040, −0.002)	0.0047	−6.571	3.40 × 10^−9^	*ASB10*
cg00340919	5	94879974	−0.0517 (−0.069, −0.034)	0.0089	−5.836	8.74 × 10^−8^	*TTC37*

*^∗^Base position-Human Genome version 19 (GRCh37/hg19).*

Regression analyses of hcy measures for both AA and EA (hcy, POST, DeltaPost) identified 56 CpG sites that met the suggestive threshold (**Supplementary Figures S1**–**S6** and **Supplementary Table S2**). For all hcy related analyses, the two most significant associations were observed at cg08657886 (AA POST; *p* = 3.26 × 10^−7^) and cg21649277 (EA POST; *p* = 4.69 × 10^−7^), both located on chromosome 19. Methylation locus cg08657886 was located within the promoter region of the Family with sequence similarity 32, member A (*FAM32A*) gene, while cg21649277 is located in the first intron of the arrestin domain containing 2 gene, *ARRDC2* (**Supplementary Table S2**). Suggestive evidence of association for the POST phenotype at cg14818960, located within the first intron of the folate receptor 1 gene, *FOLR1* (*p* = 9.66 × 10^−6^) is also notable, given this gene’s function in the FOCM pathway (**Supplementary Table S2**).

Gene ontology analysis using GOrilla, identified 22 terms with suggestive association (*p* ≤ 1.0 × 10^−3^) using the top 86 CpG sites from the PNS regression (**Supplementary Table S3**). Sulfur compound binding (GO:1901681) was the most significant GO term (*p* = 5.01 × 10^−5^), followed by heparin binding (GO:0008201; *p* = 5.38 × 10^−5^). The genes *ASB10* and *TTC37* were both associated at the suggestive threshold with the terms signal transduction (GO:0007165) and (unknown) molecular function (GO:0003674), albeit both of these terms are broadly defined. missMethyl identified 55 suggestive GO terms at *p* ≤ 5 × 10^−3^, including proteinaceous ECM (GO:0005578, *p* = 6.15 × 10^−5^) and adenylate cyclase-inhibiting G-protein coupled receptor signaling pathway (GO:0007193, *p* = 1.20 × 10^−4^) (**Supplementary Table S4**).

## Discussion

Differential methylation analyses were performed for six phenotypes related to hcy and recurrent stroke, resulting in 140 significant or suggestive associations. Two epigenome-wide significant, differentially methylated CpG sites located within the *ASB10* and *TTC37* genes were associated with PNS in the EA subgroup. These two methylation loci were not statistically significant in the AA subset analyses for PNS (cg22812874 *p* = 0.554, β = −2.445; 95% CI = −10.682, 5.791; cg00340919 *p* = 0.397, β = 0.841; 95% CI = −1.135, 2.818).

The *ASB10* gene functions by targeting SOCS proteins and the elongin BC regulatory complex for degradation (Kile et al., 2002). There are no studies implicating this gene with stroke risk, however, a synonymous variant in *ASB10* has been associated with primary open angle glaucoma (Pasutto et al., 2012). Immunofluorescence and confocal analyses determined that *ASB10* is localized intracellularly in human TM cells (Keller et al., 2013) and that TNF-α and IL-1β up-regulate *ASB10* in these cells (Keller and Wirtz, 2017). TNF-α and IL-1β are risk factors for ischemic stroke in humans (Luheshi et al., 2011; Bokhari et al., 2014) and result in neurotoxicity in rats after an ischemic event (Clausen et al., 2008). As previously mentioned, the associated methylation locus cg22812874 is located in a TFBS for the CTCF protein as predicted using ORegAnno data from UCSC Genome Browser (Kent et al., 2002; Montgomery et al., 2006; Griffith et al., 2008). This protein is ubiquitously expressed and has been described traditionally as a transcriptional repressor (Lobanenkov et al., 1990; Holwerda and de Laat, 2013). Additionally, CTCF can act as an insulator when positioned between an enhancer and promoter. The insulator activity blocks signaling between the enhancer and promoter and prevents transcriptional activation (Bell et al., 1999). CTCF was shown to be essential in cardiogenesis, as well as in the mediation of cardiomyocyte differentiation. Inactivation of this transcription factor causes severe cardiac defects and death in embryonic development (Gomez-Velazquez et al., 2017). The ASB10 association is likely specific to populations of European decent, as our AA analyses showed no evidence for association. A recent study of ischemic cardiomyopathy in a Spanish population detected significant differential DNA methylation patterns for another member of the ASB family (ASB1 gene), and further highlighted the potential role of DNA methylation of ASB family members for stroke and cardiovascular phenotypes (Ortega et al., 2018).

*TTC37* encodes a protein with twenty tetratricopeptide repeats, which is highly expressed in vascular tissues (Fabre et al., 2011) and has been implicated in THES. Symptoms of THES include platelet abnormalities, such as large platelet sizes and thrombocytosis (Hartley et al., 2010), which are risk factors for stroke (Arboix et al., 1995; Posfai et al., 2014; Kato et al., 2015). While the associated methylation locus cg00340919 is not directly located in a regulatory element, it is adjacent to the SNP rs73777218. Combined eQTL analyses from GTEx suggests that rs73777218 is associated with the expression of pseudogene CTD-2538A21.1 in brain-cerebellum tissues (GTEx Consortium, 2013, 2017 Hormozdiari et al., 2014; Ongen et al., 2016). Although limited knowledge is available on the function of this particular pseudogene, it is located within intron 7 of the FAM81B gene. Expression of FAM81B is related to progression free survival of estrogen positive breast cancer when treated with aromatase inhibitors (Ramirez-Ardila et al., 2016). Although debatable, aromatase inhibitors are thought to increase risk of coronary heart disease and stroke in women (Chlebowski et al., 2006).

The most differentially methylated loci in the hcy analyses were cg08657886 and cg21649277. cg08657886 is located within the promoter region of *FAM32A*, a housekeeping gene whose protein may function in inducing G2 arrest and apoptosis (The UniProt Consortium, 2017). cg21649277 is located in the first intron of *ARRDC2*, whose protein is a member of the arrestin-related trafficking (ART) family of proteins (Rauch and Martin-Serrano, 2011). ART proteins functions as regulators of G protein-mediated signaling. The most notable locus that reached suggestive significance with functional implications in FOCM or cerebral diseases was cg14818960. This locus is located within *FOLR1*, the gene encoding the folate receptor-α protein, which regulates folate transport into cells. Folate receptor-α is found within cell membranes, where it binds to 5-MTHF (Steinfeld et al., 2009). Variants in *FOLR1* have been independently associated with cerebral folate transport deficiency and brain abnormalities caused by the lack of folate (Steinfeld et al., 2009).

GO analyses, using GOrilla and missMethyl, identified 22 and 55 terms, respectively, that reached a suggestive threshold. The most statistically significant term across both packages was sulfur compound binding including but not limited to organosulfur compounds diallyl sulfide and allyl mercaptan. These compounds have been recognized to decrease total cholesterol, triglycerides, thrombosis, and lower blood pressure (Warshafsky et al., 1993; Kris-Etherton et al., 2002), all of which are risk factors of cardiovascular disease.

Both GOrilla and missMethyl identified heparin binding as a molecular function with a suggestive association. Heparin, a commonly prescribed anticoagulant, is used in ischemic stroke prevention. Studies focusing on the efficacy and safety of heparin alone or in combination therapies have demonstrated that heparin is safe and effective in ischemic attacks (Yi et al., 2014; Dluha et al., 2016); however, novel therapeutics are becoming more promising and could soon be implemented more regularly in favor to traditional heparin treatment (Douketis et al., 2015; Han et al., 2015). The ontology terms that were suggestively associated with *ASB10* and *TTC37* included signal transduction. Signal transduction mediators have been reported in brain ischemia injury and depending on the mediator, have both neuroprotective and neurotoxic effects (Jin et al., 2013).

missMethyl enrichment identified proteinaceous ECM and G-protein-coupled receptors as suggestive terms. The proteinaceous ECM is a layer that consists predominately of proteins and glycosaminoglycans (Binns et al., 2009; QuickGO, 2017). ECM composition was reported to be altered upon BBB disruption, which directly affects neurological disease progression (Baeten and Akassoglou, 2011). Degradation of these ECM components by MMPs has been reported (Mott and Werb, 2004; Cai et al., 2015). Matrix metalloproteinase-9 (MMP-9) has been associated with ischemic stroke progression in mouse brain models (Zhao et al., 2006). MMP-9 upregulated in the first 3 days after ischemic stroke contributed to BBB permeability and brain inflammation. However, elevated levels of MMP-9 on days 7–14 post-stroke played a beneficial role in angiogenesis and brain recovery (Zhao et al., 2006). Therefore, strategies that modulate MMP function in regulation of ECM components can be utilized for ischemic stroke recovery. In addition, G-protein-coupled receptors have been implicated in neuronal function and protection in human studies, cell-culture, and animal models (Martin et al., 2005). Possible roles for class II G-protein receptors have been revealed in the pathogenesis of neurological and neurodegenerative conditions (Martin et al., 2005).

This study has a number of strengths and limitations. The VISP clinical trial included a mixture of both AA and EA participants and was well-phenotyped from a clinical perspective. Although specific stroke subtypes were not included, based on the inclusion/exclusion criteria, the strokes likely represented a homogenous, small-vessel disease group. Recurrent stroke is a grossly understudied phenotype; therefore the inclusion of recurrent stroke phenotypes is a strength of this study. However, no public datasets are available with both epigenetic (DNA methylation) data and stroke recurrence phenotypes. This limitation prohibits us from validating our findings, yet further emphasizes the need for more studies focused on these traits/datasets. The use of the commercially available Illumina arrays allowed the entire epigenome to be evaluated with confidence and reproducibility.

The VISP clinical trial had a randomized, controlled, double-blind experimental design, and thus does not represent a population sample. Study entry required hcy levels in the top quartile, while the methylation subset included VISP participants with hcy levels at the extremes. All subjects experienced an ischemic stroke within 120 days of enrollment, thus our sample is skewed toward an older age distribution with hypertension and elevated body mass indexes. Despite its relevance for a stroke recurrence phenotype, our hcy results may not be generalizable. We are unable to determine the timing of the epigenetic changes relative to the phenotypes investigated, thus we can only establish association and not causation.

DNA from VISP participants was extracted from whole blood samples. Although not optimal due to cellular heterogeneity, whole blood provides a valuable resource that is potentially available for replication studies. To minimize confounding effects due to cell heterogeneity in whole blood, cell-type proportions were estimated based on methylation signatures and were included as covariates in the regression models. The Houseman method, as implemented in minfi, is generally regarded as the gold standard for cell proportion estimations in DNA methylation studies using whole blood. Alternative programs exist that are capable of generating cell estimations and corresponding cell proportions. Using MeDeCom (Lutsik et al., 2017), we compared latent DNA methylation components (LMCs) with estimated cell proportions from minfi (**Supplementary Figure S12** and **Supplementary Table S5**). Monocytes and NK cells are not fully captured in the LMC estimations. Monocytes are thought to play a major role in ischemic stroke pathology (ElAli and LeBlanc, 2016), therefore the minfi cell proportion estimations which account for monocyte proportions were considered most biologically appropriate, and were utilized in our analysis. Despite having limited power, statistically significant associations between two CpG sites and PNS in EA were detected, but *larger*, independent *sample sizes* will be needed to confirm *and validate* our results. Overall, these analyses present a foundation for further epigenetic investigations of novel loci in stroke-susceptible populations.

## Data Availability Statement

The datasets used and analyzed during this study are available from the corresponding author on reasonable request.

## Author Contributions

NDA contributed in data analysis, interpretation of the data, and drafting of the manuscript. W-MC contributed in data analysis, interpretation, and revising of the manuscript. MB contributed in data manipulation, quality control and analysis, and revising of the manuscript. SW contributed in the study design and revising of the manuscript. MS and BW contributed in overall conception, design and interpretation of the data, and revising of the manuscript. KK contributed in overall conception, design and interpretation of the data, drafting and revising of the manuscript. All authors read and approved the final manuscript.

## Conflict of Interest Statement

The authors declare that the research was conducted in the absence of any commercial or financial relationships that could be construed as a potential conflict of interest.

## Abbreviations

5-MTHF: 5-methyl-tetrahydrofolate
AA: African American
ARRDC2: arrestin domain containing 2 gene
ASB10: Ankyrin repeat and SOCs box containing 10
BBB: blood–brain barrier
BCD: bisulfite-converted DNA
DeltaPost: Absolute difference of post-methionine load test and baseline homocysteine levels
EA: European American
ECM: extracellular matrix
eQTL: expression quantitative trait loci
FAM32A: Family with sequence similarity 32, member A gene
FOCM: folate-mediated one-carbon metabolism
FOLR1: folate receptor alpha
GO: gene ontology
GTEx: Genotype-Tissue Expression
GWAS: genome-wide association study
Hcy: Homocysteine
HCY: Measured baseline homocysteine level
IL-1β: interleukin-1 beta
MMP: matrix metalloproteinases
MTHFR: Methylenetetrahydrofolate reductase
NDCI: non-disabling cerebral infarction
PCA: principal component analysis
PNS: previous number of strokes
POST: Post-methionine load test homocysteine level
Recurrent Ever: Recurrent stroke status prior to or during VISP trial
SNP: single nucleotide polymorphism
SOCs: suppressor of cytokine signaling
TFBS: transcription factor binding site
THES: trichohepatoenteric syndrome
TM cells: trabecular meshwork cells
TNF-α: tumor necrosis factor alpha
TTC37: tetratricopeptide repeat domain 37 gene
VISP: Vitamin Intervention for Stroke Prevention
VISP Recurrent: Recurrent stroke during the VISP trial.
