# Supplementary material for: Epigenome-Wide Analyses Identify Two Novel Associations With Recurrent Stroke in the Vitamin Intervention for Stroke Prevention Clinical Trial
Source: Front Genet. 2018 Sep 6;9:358. doi: 10.3389/fgene.2018.00358 (PMC6135883; doi:10.3389/fgene.2018.00358)
Supplement: Supplementary file 1 [file Table_1.DOCX]

***Supplementary Material***

**Epigenome-wide analyses identify two novel associations with recurrent stroke in the Vitamin Intervention for Stroke Prevention clinical trial.**

Nicole M. Davis Armstrong^1^, Wei-Min Chen^2,3^, Michael S. Brewer^1^, Stephen R. Williams^4^, Michèle M. Sale^2,3^, Bradford B. Worrall ^3,4^, and Keith L. Keene^1,5*^

^1^ Department of Biology, East Carolina University, Greenville, NC, USA

^2^ Center for Public Health Genomics, University of Virginia, Charlottesville, VA, USA

^3^Department of Public Health Sciences, University of Virginia, Charlottesville, VA, USA

^4^Department of Neurology, University of Virginia, Charlottesville, VA, USA

^5^Center for Health Disparities, East Carolina University, Greenville, NC, USA

**Please address correspondence to:**

Dr. Keith L. Keene

East Carolina University

Department of Biology; Center for Health Disparities
[keenek@ecu.edu](mailto:bbw9r@virginia.edu)

(252) 328-1838

**Number of Supplemental Tables: 5**

**Number of Supplemental Figures: 12**

**Supplementary Table 1: Differentially methylated loci for recurrent stroke risk at 5.0x10^-5^ significance level**

| **Analysis Group** | **Locus** | **CHR** | **BP** | **Coefficient** | **Lower CI** | **Upper CI** | **SE** | **t-stat** | **P-value** | **Gene (upstream)** |
| --- | --- | --- | --- | --- | --- | --- | --- | --- | --- | --- |
| EA PNS | cg22812874 | 7 | 150872892 | -0.0308 | -0.0402 | -0.0215 | 0.0047 | -6.5708 | 3.40 x10-9 | *ASB10* |
| EA PNS | cg00340919 | 5 | 94879974 | -0.0517 | -0.0693 | -0.0341 | 0.0089 | -5.8364 | 8.74 x10-8 | *TTC37* |
| EA PNS | cg08563299 | 19 | 16922592 | -0.0443 | -0.0605 | -0.0281 | 0.0082 | -5.4277 | 4.98 x10-7 | *NWD1* |
| EA PNS | cg00020622 | 2 | 144694680 | 0.0060 | 0.0038 | 0.0082 | 0.0011 | 5.3527 | 6.82 x10-7 | *(GTDC1)* |
| EA PNS | cg15493607 | 14 | 88738195 | -0.0188 | -0.0260 | -0.0116 | 0.0036 | -5.1751 | 1.42 x10-6 | *KCNK10* |
| EA PNS | cg19869139 | 1 | 29138077 | -0.0267 | -0.0370 | -0.0164 | 0.0052 | -5.1454 | 1.61 x10-6 | *OPRD1* |
| EA PNS | cg24037380 | 17 | 73727879 | -0.0162 | -0.0227 | -0.0098 | 0.0032 | -5.0428 | 2.44 x10-6 | *ITGB4* |
| EA PNS | cg15976416 | 1 | 1280157 | -0.0141 | -0.0197 | -0.0085 | 0.0028 | -5.0134 | 2.75 x10-6 | *DVL1* |
| EA PNS | cg07010582 | 6 | 32046308 | -0.0261 | -0.0366 | -0.0156 | 0.0053 | -4.9323 | 3.81 x10-6 | *TNXB* |
| EA PNS | cg16086416 | 5 | 3339474 | -0.0240 | -0.0337 | -0.0143 | 0.0049 | -4.8979 | 4.37 x10-6 | *(IRX1)* |
| EA PNS | cg00810334 | 13 | 114324491 | -0.0305 | -0.0429 | -0.0181 | 0.0062 | -4.894 | 4.44 x10-6 | *GRK1* |
| EA PNS | cg11241413 | 5 | 33970305 | -0.0502 | -0.0706 | -0.0297 | 0.0103 | -4.8819 | 4.66 x10-6 | *SLC45A2* |
| EA PNS | cg20424709 | 10 | 37940673 | -0.0419 | -0.0591 | -0.0248 | 0.0086 | -4.8525 | 5.23 x10-6 | *(ZNF248)* |
| EA PNS | cg12452608 | 3 | 61179015 | -0.0131 | -0.0185 | -0.0077 | 0.0027 | -4.8226 | 5.90 x10-6 | *FHIT* |
| EA PNS | cg24461508 | 19 | 40505779 | -0.0165 | -0.0233 | -0.0097 | 0.0034 | -4.8213 | 5.93 x10-6 | *ZNF546* |
| EA PNS | cg01741999 | 2 | 219137824 | -0.0225 | -0.0319 | -0.0132 | 0.0047 | -4.7994 | 6.46 x10-6 | *PNKD* |
| EA PNS | cg21607141 | 4 | 119722917 | -0.0255 | -0.0362 | -0.0149 | 0.0053 | -4.7742 | 7.14 x10-6 | *SEC24D* |
| EA PNS | cg09967479 | 12 | 133010684 | -0.0283 | -0.0400 | -0.0165 | 0.0059 | -4.7723 | 7.19 x10-6 | *(FBRSL1)* |
| EA PNS | cg21204139 | 1 | 64669336 | -0.0356 | -0.0504 | -0.0207 | 0.0075 | -4.7567 | 7.65 x10-6 | *UBE2U* |
| EA PNS | cg26542412 | 1 | 92350568 | -0.0128 | -0.0183 | -0.0074 | 0.0027 | -4.7179 | 8.91 x10-6 | *TGFBR3* |
| EA PNS | cg26836573 | 5 | 43280399 | -0.0247 | -0.0351 | -0.0143 | 0.0052 | -4.7154 | 8.99 x10-6 | *MGC42105* |
| EA PNS | cg08371391 | 20 | 19739935 | -0.0422 | -0.0600 | -0.0243 | 0.0090 | -4.693 | 9.82 x10-6 | *(RIN2)* |
| EA PNS | cg22093885 | 2 | 189471198 | -0.0359 | -0.0511 | -0.0207 | 0.0077 | -4.6841 | 1.02 x10-5 | *(GULP1)* |
| EA PNS | cg25108325 | 1 | 64669472 | -0.0336 | -0.0479 | -0.0194 | 0.0072 | -4.68 | 1.03 x10-5 | *UBE2U* |
| EA PNS | cg01075918 | 18 | 9912834 | -0.0118 | -0.0169 | -0.0068 | 0.0025 | -4.657 | 1.13 x10-5 | *VAPA* |
| EA PNS | cg09474101 | 2 | 105279258 | -0.0103 | -0.0147 | -0.0059 | 0.0022 | -4.6548 | 1.14 x10-5 | *(POU3F3)* |
| EA PNS | cg20539307 | 8 | 11715284 | -0.0152 | -0.0217 | -0.0087 | 0.0033 | -4.6554 | 1.14 x10-5 | *CTSB* |
| EA PNS | cg12851161 | 13 | 22298502 | -0.0170 | -0.0243 | -0.0097 | 0.0037 | -4.6494 | 1.16 x10-5 | *(FGF9)* |
| EA PNS | cg20756175 | 4 | 95762633 | -0.0117 | -0.0168 | -0.0067 | 0.0025 | -4.639 | 1.21 x10-5 | *BMPR1B* |
| EA PNS | cg05302953 | 18 | 43654662 | -0.0214 | -0.0305 | -0.0122 | 0.0046 | -4.6173 | 1.32 x10-5 | *(PSTPIP2)* |
| EA PNS | cg02134194 | 16 | 1162029 | -0.0350 | -0.0502 | -0.0198 | 0.0076 | -4.5877 | 1.48 x10-5 | *(C1QTNF8)* |
| EA PNS | cg02696670 | 1 | 64669384 | -0.0279 | -0.0400 | -0.0158 | 0.0061 | -4.5781 | 1.53 x10-5 | *UBE2U* |
| EA PNS | cg24479785 | 11 | 1029029 | -0.0136 | -0.0195 | -0.0077 | 0.0030 | -4.5712 | 1.58 x10-5 | *MUC6* |
| EA PNS | cg14925296 | 19 | 39995913 | -0.0099 | -0.0142 | -0.0056 | 0.0022 | -4.556 | 1.67 x10-5 | *DLL3* |
| EA PNS | cg00481457 | 18 | 21269165 | -0.0058 | -0.0084 | -0.0033 | 0.0013 | -4.553 | 1.69 x10-5 | *LAMA3* |
| EA PNS | cg07664976 | 15 | 25344488 | -0.0398 | -0.0572 | -0.0224 | 0.0088 | -4.5471 | 1.73 x10-5 | *SNORD116-26* |
| EA PNS | cg01431140 | 17 | 35118924 | -0.0135 | -0.0194 | -0.0076 | 0.0030 | -4.5398 | 1.78 x10-5 | *(LHX1)* |
| EA PNS | cg04291946 | 20 | 60791310 | -0.0414 | -0.0597 | -0.0232 | 0.0092 | -4.5066 | 2.02 x10-5 | *HRH3* |
| EA PNS | cg25061969 | 8 | 11946498 | -0.0213 | -0.0307 | -0.0119 | 0.0047 | -4.4977 | 2.09 x10-5 | *ZNF705D* |
| EA PNS | cg18157379 | 3 | 67511061 | -0.0258 | -0.0372 | -0.0143 | 0.0058 | -4.4723 | 2.30 x10-5 | *SUCLG2* |
| EA PNS | cg26317111 | 1 | 64669415 | -0.0329 | -0.0475 | -0.0182 | 0.0074 | -4.4671 | 2.35 x10-5 | *UBE2U* |
| EA PNS | cg06210054 | 1 | 36173658 | -0.0266 | -0.0385 | -0.0148 | 0.0060 | -4.4633 | 2.38 x10-5 | *(PSMB2)* |
| EA PNS | cg07325827 | 17 | 21226936 | -0.0198 | -0.0286 | -0.0110 | 0.0044 | -4.4598 | 2.41 x10-5 | *(KCNJ12)* |
| EA PNS | cg18136062 | 15 | 61520931 | -0.0152 | -0.0220 | -0.0084 | 0.0034 | -4.4556 | 2.45 x10-5 | *RORA* |
| EA PNS | cg20439283 | 5 | 178411249 | -0.0184 | -0.0266 | -0.0101 | 0.0041 | -4.4453 | 2.55 x10-5 | *GRM6* |
| EA PNS | cg27181253 | 1 | 113051846 | -0.0294 | -0.0426 | -0.0163 | -4.4370 | -4.4367 | 2.63 x10-5 | *WNT2B* |
| EA PNS | cg11167637 | 1 | 64669432 | -0.0308 | -0.0446 | -0.0170 | 0.0069 | -4.4365 | 2.64 x10-5 | *UBE2U* |
| EA PNS | cg11805401 | 14 | 104633638 | -0.0255 | -0.0370 | -0.0141 | 0.0058 | -4.4342 | 2.66 x10-5 | *KIF26A* |
| EA PNS | cg27234747 | 2 | 120525972 | -0.0148 | -0.0214 | -0.0081 | 0.0033 | -4.4196 | 2.81 x10-5 | *PTPN4* |
| EA PNS | cg14370595 | 11 | 66114071 | -0.0052 | -0.0075 | -0.0028 | 0.0012 | -4.4172 | 2.84 x10-5 | *BRMS;B3GNT1* |
| EA PNS | cg18924771 | 5 | 166582591 | -0.0391 | -0.0568 | -0.0215 | 0.0089 | -4.4152 | 2.86 x10-5 | *(ODZ2)* |
| EA PNS | cg10603296 | 4 | 20529748 | -0.0267 | -0.0388 | -0.0147 | 0.0061 | -4.4095 | 2.92 x10-5 | *MIR218-1;SLIT2* |
| EA PNS | cg10513866 | 5 | 139070639 | -0.0243 | -0.0352 | -0.0133 | 0.0055 | -4.4045 | 2.98 x10-5 | *(PSD2)* |
| EA PNS | cg19439894 | 2 | 92280697 | -0.0164 | -0.0238 | -0.0090 | 0.0037 | -4.4037 | 2.98 x10-5 | *(GGT8P)* |
| EA PNS | cg14587401 | 5 | 94890408 | 0.0378 | 0.0208 | 0.0549 | 0.0086 | 4.4016 | 3.01 x10-5 | *ARSK;TTC37* |
| EA PNS | cg21544475 | 20 | 48431765 | -0.0365 | -0.0530 | -0.0200 | 0.0083 | -4.4012 | 3.01 x10-5 | *SLC9A8* |
| EA PNS | cg17001652 | 4 | 78432403 | -0.0287 | -0.0417 | -0.0157 | 0.0065 | -4.3972 | 3.06 x10-5 | *CXCL13* |
| EA PNS | cg27516302 | 8 | 54162969 | -0.0337 | -0.0490 | -0.0184 | 0.0077 | -4.3775 | 3.29 x10-5 | *OPRK1* |
| EA PNS | cg08048699 | 11 | 57296305 | -0.0283 | -0.0411 | -0.0154 | 0.0065 | -4.3661 | 3.44 x10-5 | *TIMM10* |
| EA PNS | cg25436414 | 10 | 50966914 | -0.0197 | -0.0287 | -0.0107 | 0.0045 | -4.3656 | 3.45 x10-5 | *OGDHL* |
| EA PNS | cg07302938 | 7 | 12729527 | -0.0117 | -0.0171 | -0.0064 | 0.0027 | -4.3478 | 3.68 x10-5 | *ARL4A* |
| EA PNS | cg06006512 | 3 | 89221147 | -0.0196 | -0.0285 | -0.0106 | 0.0045 | -4.3438 | 3.74 x10-5 | *EPHA3* |
| EA PNS | cg25389947 | 7 | 64838786 | -0.0084 | -0.0122 | -0.0045 | 0.0019 | -4.3428 | 3.75 x10-5 | *ZNF92* |
| EA PNS | cg11280288 | 3 | 130462135 | -0.0264 | -0.0385 | -0.0143 | 0.0061 | -4.3342 | 3.87 x10-5 | *PIK3R4* |
| EA PNS | cg18199617 | 1 | 120311653 | -0.0243 | -0.0354 | -0.0131 | 0.0056 | -4.3326 | 3.90 x10-5 | *HMGCS2* |
| EA PNS | cg27625815 | 8 | 8195300 | -0.0106 | -0.0154 | -0.0057 | 0.0024 | -4.3315 | 3.91 x10-5 | *PRAGMIN* |
| EA PNS | cg12458280 | 11 | 128750088 | -0.0219 | -0.0320 | -0.0118 | 0.0051 | -4.318 | 4.12 x10-5 | *(KCNJ5)* |
| EA PNS | cg11707067 | 19 | 59073430 | -0.0144 | -0.0210 | -0.0078 | 0.0033 | -4.3134 | 4.19 x10-5 | *MZF1* |
| EA PNS | cg00365880 | 15 | 64647465 | -0.0195 | -0.0285 | -0.0105 | 0.0045 | -4.3065 | 4.30 x10-5 | *CSNK1G1* |
| EA PNS | cg13200730 | 17 | 25827857 | -0.0271 | -0.0395 | -0.0146 | 0.0063 | -4.3047 | 4.33 x10-5 | *KSR1* |
| EA PNS | cg13115444 | 11 | 66339199 | -0.0173 | -0.0253 | -0.0093 | 0.0040 | -4.3039 | 4.34 x10-5 | *(CTSF)* |
| EA PNS | cg01820765 | 16 | 85241897 | -0.0040 | -0.0059 | -0.0022 | 0.0009 | -4.3 | 4.40 x10-5 | *(FAM92B)* |
| EA PNS | cg09313587 | 19 | 3295021 | -0.0262 | -0.0383 | -0.0141 | 0.0061 | -4.298 | 4.44 x10-5 | *BRUNOL5* |
| EA PNS | cg12653146 | 1 | 25919290 | -0.0128 | -0.0187 | -0.0069 | 0.0030 | -4.2928 | 4.52 x10-5 | *(LDLRAP1)* |
| EA PNS | cg17903019 | 7 | 5552472 | -0.0464 | -0.0679 | -0.0249 | 0.0108 | -4.289 | 4.59 x10-5 | *FBXL18* |
| EA PNS | cg05755715 | 2 | 21646627 | -0.0316 | -0.0462 | -0.0169 | 0.0074 | -4.2853 | 4.65 x10-5 | *(APOB)* |
| EA PNS | cg00924575 | 5 | 76474673 | -0.0244 | -0.0357 | -0.0131 | 0.0057 | -4.2783 | 4.77 x10-5 | *(PDE8B)* |
| EA PNS | cg15781542 | 19 | 48180471 | -0.0134 | -0.0196 | -0.0072 | 0.0031 | -4.2765 | 4.81 x10-5 | *GLTSCR1* |
| EA PNS | cg00334550 | 6 | 129250794 | -0.0123 | -0.0181 | -0.0066 | 0.0029 | -4.2742 | 4.85 x10-5 | *LAMA2* |
| EA PNS | cg17347063 | 22 | 36043021 | -0.0194 | -0.0284 | -0.0104 | 0.0045 | -4.27 | 4.92 x10-5 | *APOL6* |
| AA PNS | cg01724006 | 1 | 43233072 | -0.0031 | -0.0044 | -0.0017 | 0.0007 | -4.543 | 3.33 x10-5 | *LEPRE1* |
| AA PNS | cg13603533 | 4 | 48045040 | -0.0333 | -0.0476 | -0.0190 | 0.0071 | -4.671 | 2.16 x10-5 | *(TXK)* |
| AA PNS | cg02308795 | 5 | 130599504 | 0.0028 | 0.0015 | 0.0040 | 0.0006 | 4.429 | 4.89 x10-5 | *CDC42SE2* |
| AA PNS | cg01176826 | 6 | 29720527 | 0.0111 | 0.0063 | 0.0159 | 0.0024 | 4.617 | 2.59 x10-5 | *(IFITM4P)* |

**Supplementary Table 2: Differentially methylated loci for homocysteine measures at 5.0x10^-5^ significance level**

| **Analysis Group** | **Locus** | **CHR** | **BP** | **Coefficient** | **Lower CI** | **Upper CI** | **SE** | **t-stat** | **P-value** | **Gene (upstream)** |
| --- | --- | --- | --- | --- | --- | --- | --- | --- | --- | --- |
| AA Post | cg14818960 | 11 | 71903119 | 30.0437 | 17.9061 | 42.1813 | 6.0263 | 4.985 | 9.66 x10-6 | *FOLR1* |
| EA Delta Post | cg04599026 | 7 | 96634763 | -12.0807 | -17.1641 | -6.9974 | 2.5539 | -4.73 | 9.65 x10-6 | *DLX6AS;*  *DLX6* |
| AA Post | cg25943372 | 18 | 34974300 | 29.6871 | 17.7705 | 41.6036 | 5.9165 | 5.018 | 8.67 x10-6 | *CELF4;*  *BRUNOL4* |
| EA Post | cg26312935 | 15 | 59279406 | 6.0709 | 3.5536 | 8.5881 | 1.2647 | 4.8 | 7.37 x10-6 | *RNF111* |
| AA Post | cg25128696 | 13 | 112255528 | 71.6527 | 43.3108 | 99.9946 | 14.0717 | 5.092 | 6.77 x10-6 | *(TEX29)* |
| EA hcy | cg10633966 | 2 | 66660874 | -42.9046 | -60.6876 | -25.1216 | 8.9484 | -4.795 | 6.58 x10-6 | *(MEIS1)* |
| EA Post | cg05716726 | 6 | 27343671 | -6.0935 | -8.5987 | -3.5883 | 1.2586 | -4.841 | 6.29 x10-6 | *ZNF204P* |
| AA Delta Post | cg00137652 | 12 | 66696023 | 38.9868 | 23.7633 | 54.2104 | 7.5585 | 5.158 | 5.43 x10-6 | *HELB* |
| EA Post | cg04599026 | 7 | 96634763 | -12.5769 | -17.6844 | -7.4695 | 2.5660 | -4.901 | 4.98 x10-6 | *DLX6AS;*  *DLX6* |
| EA Delta Post | cg10492999 | 16 | 3063894 | 51.5500 | 27.6499 | 75.4405 | 12.0000 | 4.294 | 4.95 x10-5 | *CLDN9* |
| AA Post | cg22908581 | 12 | 6898975 | 14.1316 | 7.7918 | 20.4714 | 3.1477 | 4.489 | 4.92 x10-5 | *CD4* |
| EA Delta Post | cg06270244 | 15 | 78933407 | -24.2560 | -35.4923 | -13.0197 | 5.6451 | -4.297 | 4.89 x10-5 | *CHRNB4* |
| EA Post | cg21649277 | 19 | 18117794 | -5.7494 | -7.8332 | -3.6655 | 1.0469 | -5.492 | 4.69 x10-7 | *ARRDC2* |
| EA Delta Post | cg07209987 | 1 | 10221203 | 95.9300 | 51.6790 | 140.1751 | 22.2300 | 4.315 | 4.58 x10-5 | *UBE4B* |
| AA Post | cg22432760 | 17 | 80829718 | 18.9518 | 10.4956 | 27.4081 | 4.1985 | 4.514 | 4.55 x10-5 | *TBCD* |
| EA Delta Post | cg11866463 | 21 | 35982790 | -1.8930 | -2.7655 | -1.0205 | 0.4383 | -4.318 | 4.52 x10-5 | *RCAN1* |
| EA Delta Post | cg06953995 | 9 | 112542779 | -12.8500 | -10.4873 | 1.2926 | 2.9730 | -4.323 | 4.45 x10-5 | *PALM2;*  *PALM2-AKAP2* |
| AA hcy | cg03198203 | 11 | 17034484 | 20.0862 | 11.0379 | 29.1344 | 4.5112 | 4.453 | 4.41 x10-5 | *PLEKHA7* |
| AA Post | cg23681001 | 1 | 53936382 | 2.7736 | 1.5412 | 4.0060 | 0.6119 | 4.533 | 4.28 x10-5 | *(DMRTB1)* |
| AA hcy | cg19388776 | 3 | 71835112 | 13.8775 | 7.6489 | 20.1061 | 3.1054 | 4.469 | 4.17 x10-5 | *PROK2* |
| EA Delta Post | cg08688063 | 2 | 10588471 | -100.6000 | -146.6813 | -54.4714 | 23.1600 | -4.342 | 4.15 x10-5 | *ODC1;*  *SNORA80B* |
| AA hcy | cg15656901 | 18 | 67873348 | 77.1280 | 42.5500 | 111.7060 | 17.2395 | 4.474 | 4.10 x10-5 | *RTTN* |
| AA hcy | cg04483720 | 6 | 30227321 | 26.4261 | 14.6255 | 38.2267 | 5.8834 | 4.492 | 3.86 x10-5 | *HLA-L* |
| EA Delta Post | cg20970845 | 5 | 140241088 | 6.5490 | 3.5612 | 9.5361 | 1.5010 | 4.363 | 3.84 x10-5 | *PCDH-alpha*  *gene cluster* |
| AA Post | cg27123691 | 15 | 76004985 | 29.8087 | 16.6675 | 42.9499 | 6.5246 | 4.569 | 3.81 x10-5 | *CSPG4* |
| AA Delta Post | cg08657886 | 19 | 16295868 | 12.2987 | 6.8799 | 17.7174 | 2.6904 | 4.571 | 3.78 x10-5 | *FAM32A* |
| EA Delta Post | cg07364638 | 15 | 90808673 | 65.2800 | 35.6116 | 94.9388 | 14.9000 | 4.38 | 3.61 x10-5 | *NGRN* |
| AA Delta Post | cg22432760 | 17 | 80829718 | 15.7692 | 8.8467 | 22.6918 | 3.4370 | 4.588 | 3.58 x10-5 | *TBCD* |
| AA hcy | cg00649480 | 5 | 3593413 | 15.4259 | 8.5964 | 22.2553 | 3.4049 | 4.53 | 3.38 x10-5 | *(IRX1)* |
| AA Delta Post | cg09858281 | 8 | 142456070 | 18.1227 | 10.1984 | 26.0470 | 3.9344 | 4.606 | 3.37 x10-5 | *FLJ43860* |
| AA Post | cg06400204 | 11 | 71902997 | 27.1213 | 15.2773 | 38.9654 | 5.8806 | 4.612 | 3.31 x10-5 | *FOLR1* |
| EA Post | cg02032606 | 17 | 48049221 | -55.8843 | -81.1404 | -30.6281 | 12.6887 | -4.404 | 3.30 x10-5 | *DLX4* |
| AA Post | cg08657886 | 19 | 16295868 | 17.6735 | 11.7281 | 23.6189 | 2.9519 | 5.987 | 3.26 x10-7 | *FAM32A* |
| AA hcy | cg27237508 | 15 | 29864169 | 25.0451 | 13.9885 | 36.1018 | 5.5125 | 4.543 | 3.24 x10-5 | *FAM189A1* |
| AA hcy | cg17757575 | 17 | 34068891 | 15.3300 | 8.5629 | 22.1016 | 3.3750 | 4.543 | 3.24 x10-5 | *RASL10B* |
| AA hcy | cg08013793 | 3 | 49905354 | 23.9100 | 13.3574 | 34.4656 | 5.2620 | 4.544 | 3.23 x10-5 | *CAMKV* |
| AA Delta Post | cg18603483 | 14 | 101236070 | 23.0646 | 13.0152 | 33.1141 | 4.9895 | 4.623 | 3.19 x10-5 | *(DLK1)* |
| AA Delta Post | cg22918043 | 22 | 51001387 | 4.9512 | 2.8066 | 7.0958 | 1.0648 | 4.65 | 2.92 x10-5 | *C22orf41* |
| EA hcy | cg23887839 | 1 | 12606996 | -2.7474 | -3.9838 | -1.5110 | 0.6221 | -4.416 | 2.85 x10-5 | *(DHRS3)* |
| AA Delta Post | cg18942715 | 12 | 56510430 | 43.7471 | 24.8948 | 62.5994 | 9.3602 | 4.674 | 2.70 x10-5 | *RPL41* |
| EA Post | cg07323648 | 17 | 35291127 | -8.3355 | -12.0348 | -4.6361 | 1.8585 | -4.485 | 2.44 x10-5 | *(LHX1)* |
| EA Delta Post | cg04833898 | 16 | 2012373 | 24.3823 | 13.6151 | 35.1494 | 5.4094 | 4.507 | 2.25 x10-5 | *SNORA10;*  *RPS2* |
| EA Delta Post | cg08091147 | 19 | 1676011 | -38.2900 | -53.1928 | -23.3841 | 7.4880 | -5.113 | 2.16 x10-6 | *(TCF3)* |
| EA Post | cg08890994 | 15 | 101860935 | -18.1835 | -26.1753 | -10.1917 | 4.0151 | -4.529 | 2.07 x10-5 | *PCSK6* |
| EA Post | cg16521032 | 12 | 57587714 | 70.6402 | 39.5921 | 101.6884 | 15.5986 | 4.529 | 2.07 x10-5 | *MIR1228;*  *LRP1* |
| EA hcy | cg22550299 | 6 | 169654842 | -8.0902 | -11.6561 | -4.5243 | 1.7944 | -4.509 | 2.00 x10-5 | *THBS2* |
| AA hcy | cg00825173 | 13 | 21140752 | -71.3267 | -101.8640 | -40.7894 | 15.2249 | -4.685 | 1.99 x10-5 | *IFT88* |
| EA Delta Post | cg00515457 | 19 | 35396203 | -15.9379 | -22.9228 | -8.9529 | 3.5092 | -4.542 | 1.97 x10-5 | *(ZNF30)* |
| EA hcy | cg03595538 | 19 | 5720731 | 146.4794 | 82.1947 | 210.7641 | 32.3479 | 4.528 | 1.86 x10-5 | *TMEM146;*  *LONP1* |
| AA hcy | cg00767222 | 19 | 36169244 | 18.2819 | 10.5205 | 26.0433 | 3.8696 | 4.725 | 1.74 x10-5 | *UPK1A* |
| EA Delta Post | cg21649277 | 19 | 18117794 | -4.9670 | -7.1145 | -2.8195 | 1.0789 | -4.604 | 1.56 x10-5 | *ARRDC2* |
| AA Delta Post | cg03084648 | 8 | 23429119 | 17.6518 | 10.3313 | 24.9724 | 3.6347 | 4.857 | 1.48 x10-5 | *SLC25A37* |
| AA Delta Post | cg25943372 | 18 | 34974300 | 23.9590 | 14.0499 | 33.8682 | 4.9199 | 4.87 | 1.42 x10-5 | *CELF4;*  *BRUNOL4* |
| AA Delta Post | cg25128696 | 13 | 112255528 | 57.4481 | 33.7896 | 81.1065 | 11.7464 | 4.891 | 1.32 x10-5 | *(C13orf16)* |
| AA hcy | cg00870514 | 17 | 29717946 | 14.5282 | 8.5139 | 20.5425 | 2.9986 | 4.845 | 1.14 x10-5 | *RAB11FIP4* |
| EA Post | cg22280258 | 17 | 4545157 | -13.1587 | -18.7400 | -7.5774 | 2.8040 | -4.693 | 1.11 x10-5 | *ALOX15* |

**Supplementary Table 3: GOrilla gene ontology term enrichment analysis (suggestive threshold 1.0x10^-3^)**

| **Ontology*** | **GO Term** | **Description** | **P-value** | **Enrichment** | **Number of genes associated with term (b)** |
| --- | --- | --- | --- | --- | --- |
| MF | GO:1901681 | sulfur compound binding | 5.01x10-5 | 7.26 | 7 |
| MF | GO:0008201 | heparin binding | 5.38 x10-5 | 9.02 | 6 |
| BP | GO:0035295 | tube development | 1.01 x10-4 | 8.05 | 6 |
| BP | GO:0021871 | forebrain regionalization | 1.04 x10-4 | 118.79 | 2 |
| BP | GO:0045995 | regulation of embryonic development | 1.34 x10-4 | 10.15 | 5 |
| MF | GO:0005539 | glycosaminoglycan binding | 2.43 x10-4 | 6.85 | 6 |
| MF | GO:0004672 | protein kinase activity | 2.91 x10-4 | 3.78 | 10 |
| BP | GO:0007165 | signal transduction | 3.23 x10-4 | 1.74 | 33 |
| BP | GO:0007411 | axon guidance | 3.36 x10-4 | 6.45 | 6 |
| BP | GO:0097485 | neuron projection guidance | 3.53 x10-4 | 6.39 | 6 |
| MF | GO:0043168 | anion binding | 3.74 x10-4 | 2.07 | 23 |
| BP | GO:0038003 | opioid receptor signaling pathway | 4.82 x10-4 | 59.39 | 2 |
| MF | GO:0004985 | opioid receptor activity | 4.82 x10-4 | 59.39 | 2 |
| MF | GO:0097367 | carbohydrate derivative binding | 5.00 x10-4 | 2.19 | 20 |
| BP | GO:0009888 | tissue development | 5.73 x10-4 | 3.8 | 9 |
| BP | GO:0021702 | cerebellar Purkinje cell differentiation | 6.18 x10-4 | 52.8 | 2 |
| MF | GO:0005024 | transforming growth factor beta-activated receptor activity | 6.18 x10-4 | 52.8 | 2 |
| MF | GO:0003674 | Molecular function | 6.64 x10-4 | 1.1 | 76 |
| MF | GO:0015467 | G-protein activated inward rectifier potassium channel activity | 7.70 x10-4 | 47.52 | 2 |
| BP | GO:0032501 | multicellular organismal process | 8.12 x10-4 | 1.85 | 26 |
| BP | GO:0031581 | hemidesmosome assembly | 9.38 x10-4 | 43.2 | 2 |
| BP | GO:0006928 | movement of cell or subcellular component | 9.62 x10-4 | 2.55 | 14 |

*MF: molecular function BP: biological process

**Supplementary Table 4: missMethyl gene ontology term enrichment analysis (suggestive threshold 5.0x10^-3^)**

| **Ontology*** | **GO Term** | **Description** | **P-value** | **DE** | **Number of genes associated with term (N)** |
| --- | --- | --- | --- | --- | --- |
| CC | GO:0005578 | proteinaceous extracellular matrix | 6.15 x10-5 | 8 | 358 |
| BP | GO:0007193 | adenylate cyclase-inhibiting G-protein coupled receptor signaling pathway | 1.20 x10-4 | 4 | 68 |
| CC | GO:0031012 | extracellular matrix | 1.71 x10-4 | 8 | 421 |
| MF | GO:0004985 | opioid receptor activity | 3.54 x10-4 | 2 | 8 |
| BP | GO:0038003 | opioid receptor signaling pathway | 6.08 x10-4 | 2 | 10 |
| BP | GO:0050877 | neurological system process | 6.13 x10-4 | 11 | 1230 |
| MF | GO:0001948 | glycoprotein binding | 6.43 x10-4 | 4 | 101 |
| BP | GO:0009060 | aerobic respiration | 6.76 x10-4 | 3 | 61 |
| MF | GO:0005024 | transforming growth factor beta-activated receptor activity | 7.71 x10-4 | 2 | 10 |
| MF | GO:0097367 | carbohydrate derivative binding | 8.46 x10-4 | 17 | 2185 |
| BP | GO:0031581 | hemidesmosome assembly | 1.05 x10-3 | 2 | 12 |
| BP | GO:0007188 | adenylate cyclase-modulating G-protein coupled receptor signaling pathway | 1.22 x10-3 | 4 | 143 |
| CC | GO:0071561 | nucleus-vacuole junction | 1.24 x10-3 | 1 | 1 |
| MF | GO:1901681 | sulfur compound binding | 1.30 x10-3 | 5 | 230 |
| BP | GO:0002518 | lymphocyte chemotaxis across high endothelial venule | 1.31 x10-3 | 1 | 1 |
| BP | GO:0035769 | B cell chemotaxis across high endothelial venule | 1.31 x10-3 | 1 | 1 |
| BP | GO:1904848 | negative regulation of cell chemotaxis to fibroblast growth factor | 1.31 x10-3 | 1 | 1 |
| BP | GO:2000545 | negative regulation of endothelial cell chemotaxis to fibroblast growth factor | 1.31 x10-3 | 1 | 1 |
| MF | GO:0031724 | CXCR5 chemokine receptor binding | 1.31 x10-3 | 1 | 1 |
| MF | GO:0042923 | neuropeptide binding | 1.51 x10-3 | 2 | 22 |
| BP | GO:0045995 | regulation of embryonic development | 1.54 x10-3 | 4 | 112 |
| MF | GO:0008201 | heparin binding | 1.78 x10-3 | 4 | 153 |
| BP | GO:0043492 | ATPase activity | 1.98 x10-3 | 4 | 170 |
| MF | GO:0004672 | protein kinase activity | 2.05 x10-3 | 8 | 586 |
| MF | GO:0043168 | anion binding | 2.09 x10-3 | 18 | 2593 |
| MF | GO:0004675 | transmembrane receptor protein serine/threonine kinase activity | 2.11 x10-3 | 2 | 17 |
| MF | GO:0005518 | collagen binding | 2.17 x10-3 | 3 | 67 |
| CC | GO:0044420 | extracellular matrix component | 2.20 x10-3 | 4 | 129 |
| BP | GO:0009605 | response to external stimulus | 2.31 x10-3 | 17 | 2470 |
| BP | GO:0006099 | tricarboxylic acid cycle | 2.57 x10-3 | 2 | 29 |
| MF | GO:0015386 | potassium:proton antiporter activity | 2.78 x10-3 | 1 | 2 |
| BP | GO:0007200 | phospholipase C-activating G-protein coupled receptor signaling pathway | 2.78 x10-3 | 3 | 87 |
| BP | GO:0007411 | axon guidance | 2.94 x10-3 | 8 | 572 |
| BP | GO:0097485 | neuron projection guidance | 2.94 x10-3 | 8 | 572 |
| MF | GO:0004421 | hydroxymethylglutaryl-CoA synthase activity | 3.13 x10-3 | 1 | 2 |
| MF | GO:0031735 | CCR10 chemokine receptor binding | 3.69 x10-3 | 1 | 3 |
| BP | GO:0030198 | extracellular matrix organization | 3.78 x10-3 | 6 | 385 |
| BP | GO:0006101 | citrate metabolic process | 3.80 x10-3 | 2 | 33 |
| BP | GO:0043062 | extracellular structure organization | 3.86 x10-3 | 6 | 386 |
| BP | GO:1990708 | conditioned place preference | 4.16 x10-3 | 1 | 1 |
| MF | GO:0038048 | dynorphin receptor activity | 4.16 x10-3 | 1 | 1 |
| CC | GO:0005605 | basal lamina | 4.23 x10-3 | 2 | 22 |
| MF | GO:0017134 | fibroblast growth factor binding | 4.23 x10-3 | 2 | 23 |
| BP | GO:0072350 | tricarboxylic acid metabolic process | 4.27 x10-3 | 2 | 37 |
| BP | GO:0006464 | cellular protein modification process | 4.36 x10-3 | 22 | 3813 |
| BP | GO:0036211 | protein modification process | 4.36 x10-3 | 22 | 3813 |
| BP | GO:0006935 | chemotaxis | 4.49 x10-3 | 9 | 852 |
| BP | GO:0042330 | taxis | 4.49 x10-3 | 9 | 852 |
| MF | GO:0038046 | enkephalin receptor activity | 4.51 x10-3 | 1 | 1 |
| BP | GO:0031290 | retinal ganglion cell axon guidance | 4.56 x10-3 | 2 | 20 |
| MF | GO:0036094 | small molecule binding | 4.64 x10-3 | 17 | 2591 |
| BP | GO:0003008 | system process | 4.79 x10-3 | 13 | 1925 |
| MF | GO:0005539 | glycosaminoglycan binding | 4.86 x10-3 | 4 | 199 |
| MF | GO:0030984 | kininogen binding | 4.94 x10-3 | 1 | 3 |
| BP | GO:0042755 | eating behavior | 4.99 x10-3 | 2 | 31 |

*MF: molecular function BP: biological process CC: cellular component DE: number of differentially methylated genes


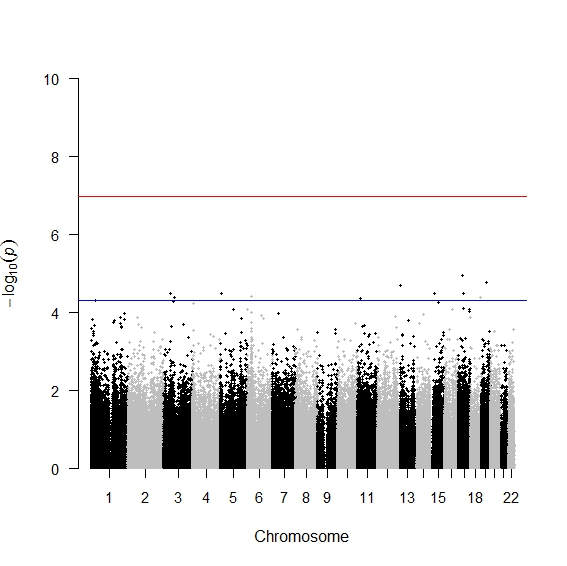


**Supplementary Figure 1**. Manhattan plot for linear regression of inverse normal transformed baseline homocysteine in African American population. Red line indicates Bonferroni-adjusted threshold of 1.055x10^-7^. Blue line indicates suggestive significance threshold of 5.0 x10^-7^.


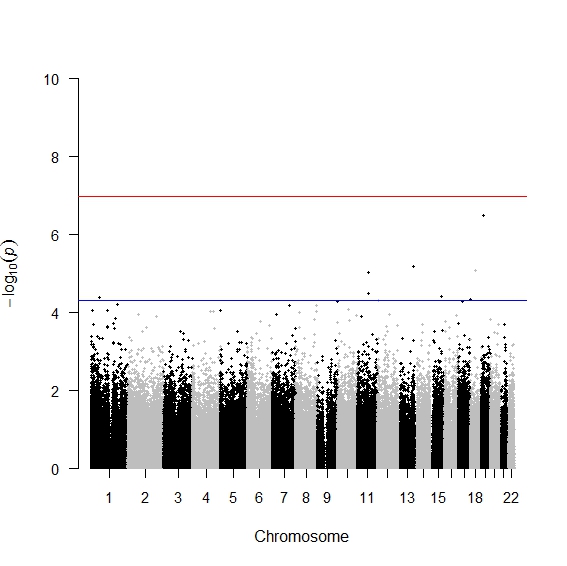


**Supplementary Figure 2**. Manhattan plot for linear regression of inverse normal transformed post-methionine homocysteine in African American population. Red line indicates Bonferroni-adjusted threshold of 1.055x10^-7^. Blue line indicates suggestive significance threshold of 5.0 x10^-7^.


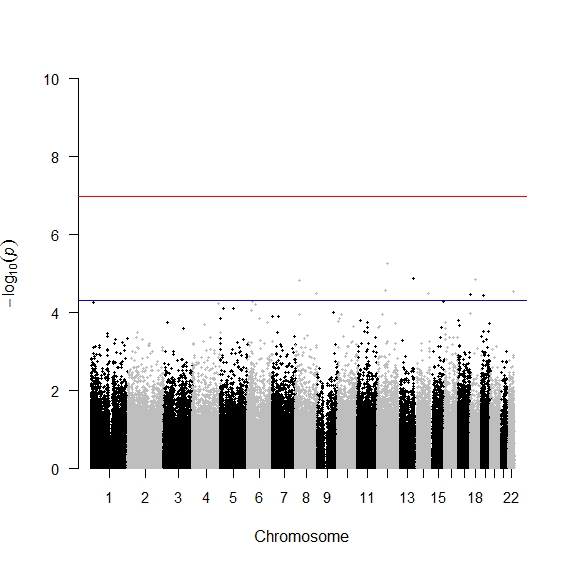


**Supplementary Figure 3.** Manhattan plot for linear regression of inverse normal transformed absolute difference of homocysteine measure (baseline homocysteine – post-methionine homocysteine) in African American population. Red line indicates Bonferroni-adjusted threshold of 1.055x10^-7^. Blue line indicates suggestive significance threshold of 5.0 x10^-7^.


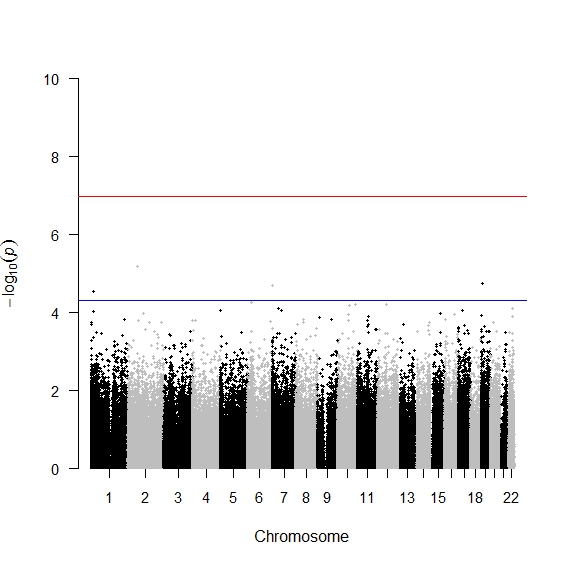


**Supplementary Figure 4.** Manhattan plot for linear regression of inverse normal transformed baseline homocysteine in European American population. Red line indicates Bonferroni-adjusted threshold of 1.055x10^-7^. Blue line indicates suggestive significance threshold of 5.0 x10^-7^.


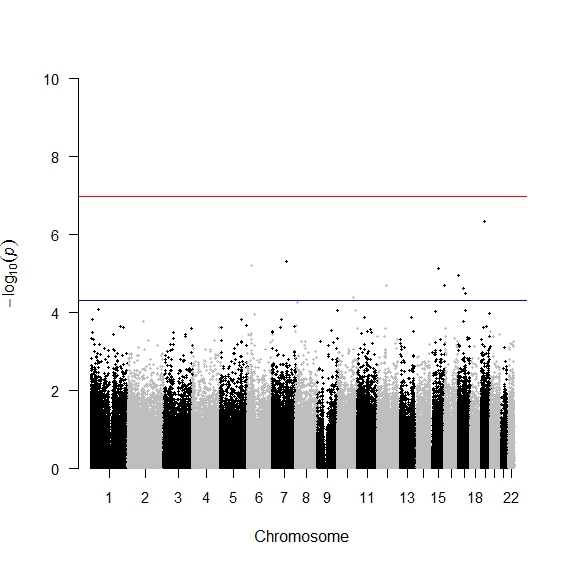


**Supplementary Figure 5.** Manhattan plot for linear regression of inverse normal transformed post-methionine homocysteine in European American population. Red line indicates Bonferroni-adjusted threshold of 1.055x10^-7^. Blue line indicates suggestive significance threshold of 5.0 x10^-7^.


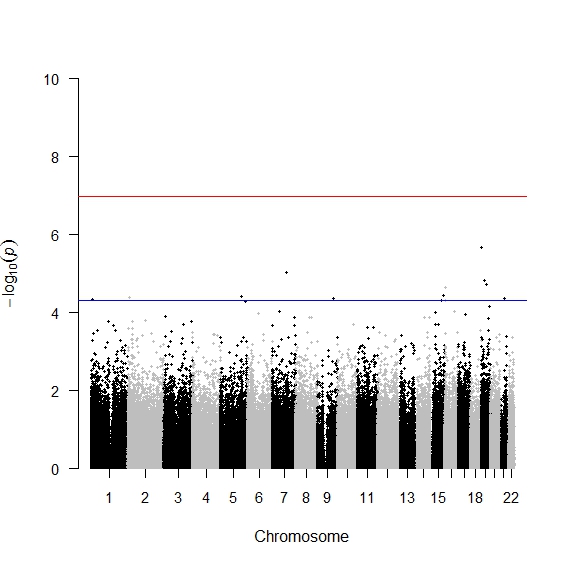


**Supplementary Figure 6.** Manhattan plot for linear regression of inverse normal transformed absolute difference of homocysteine measure (baseline homocysteine – post-methionine homocysteine) in European American population. Red line indicates Bonferroni-adjusted threshold of 1.055x10^-7^. Blue line indicates suggestive significance threshold of 5.0 x10^-7^.


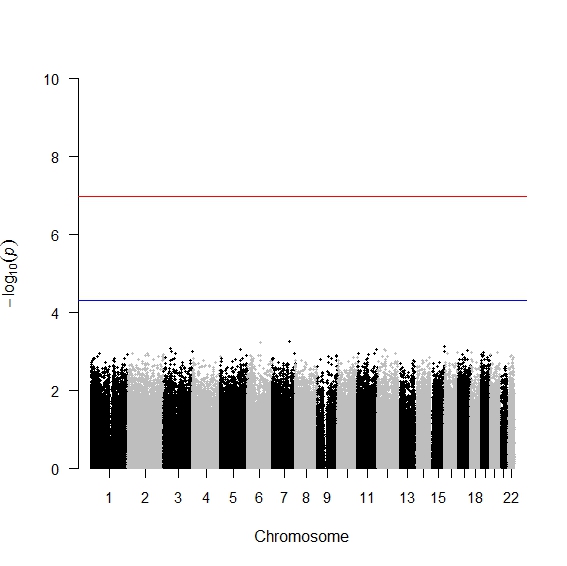


**Supplementary Figure 7.** Manhattan plot for logistic regression of recurrent stroke status (recurrent ever) in African American population. Red line indicates Bonferroni-adjusted threshold of 1.055x10^-7^. Blue line indicates suggestive significance threshold of 5.0 x10^-7^.


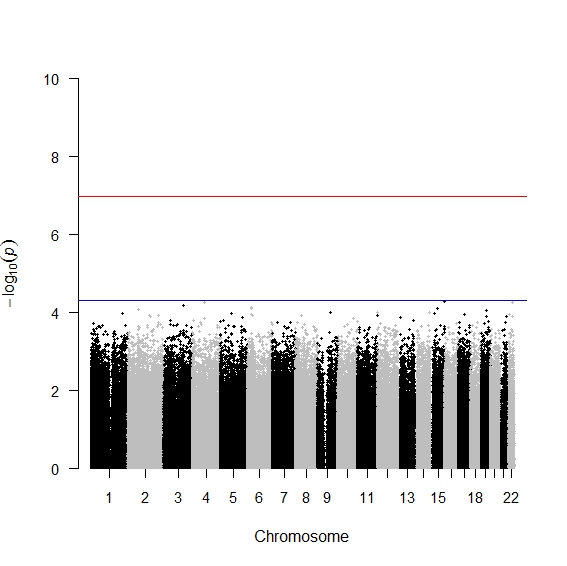


**Supplementary Figure 8.** Manhattan plot for logistic regression of recurrent stroke status (recurrent ever) in European American population. Red line indicates Bonferroni-adjusted threshold of 1.055x10^-7^. Blue line indicates suggestive significance threshold of 5.0 x10^-7^.


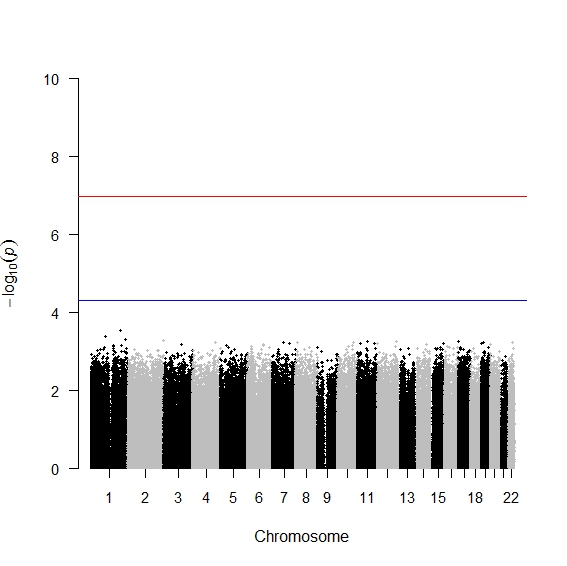


**Supplementary Figure 9.** Manhattan plot for logistic regression of recurrent stroke status during VISP trial in African American population. Red line indicates Bonferroni-adjusted threshold of 1.055x10^-7^. Blue line indicates suggestive significance threshold of 5.0 x10^-7^.


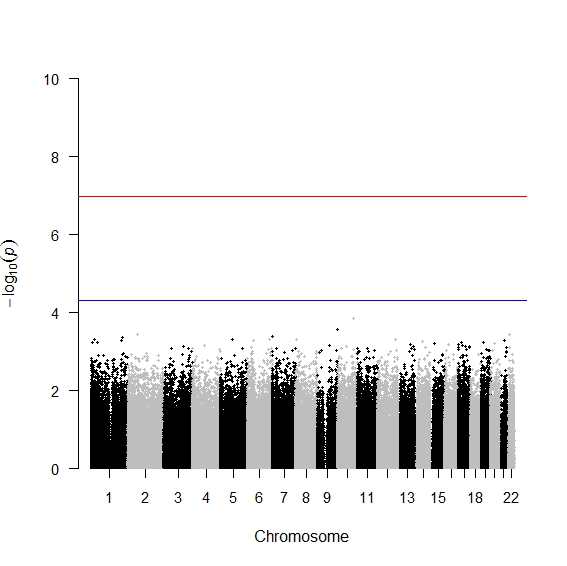


**Supplementary Figure 10.** Manhattan plot for logistic regression of recurrent stroke status during VISP trial in European American population. Red line indicates Bonferroni-adjusted threshold of 1.055x10^-7^. Blue line indicates suggestive significance threshold of 5.0 x10^-7^.


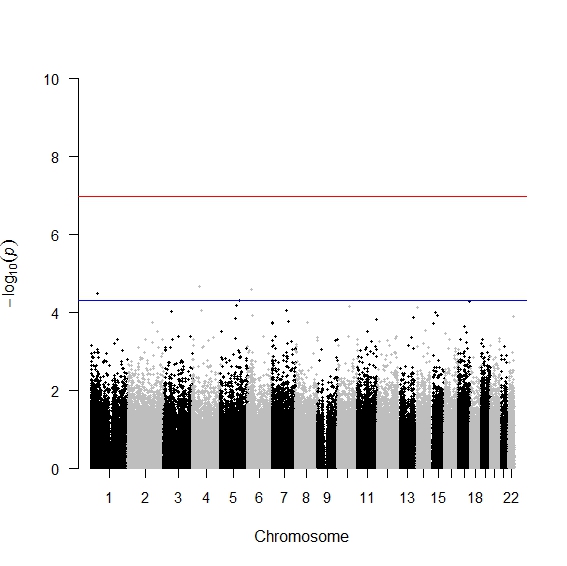


**Supplementary Figure 11.** Manhattan plot for linear regression of previous number of strokes in African American population. Red line indicates Bonferroni-adjusted threshold of 1.055x10^-7^. Blue line indicates suggestive significance threshold of 5.0 x10^-7^.


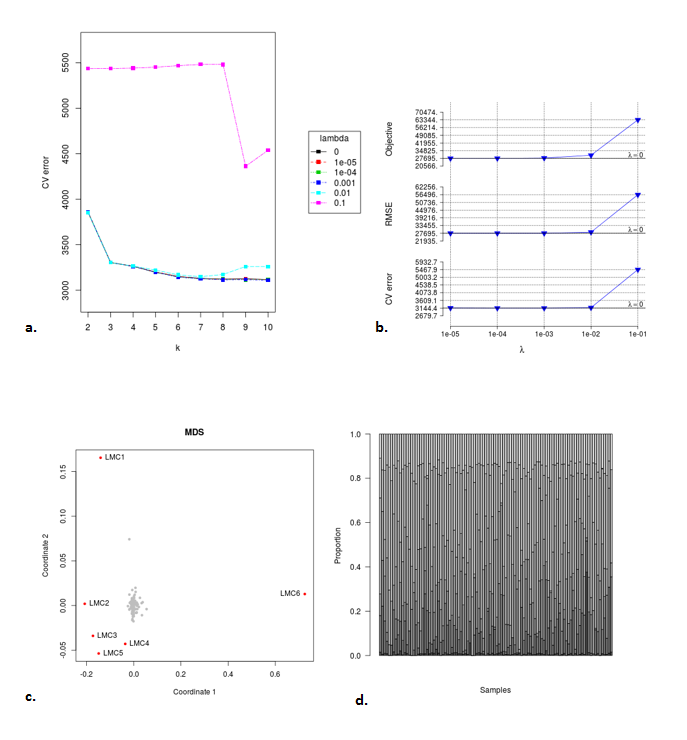


**Supplementary Figure 12.** Testing MeDeCom on EA methylation data. **A-B.** Selection of parameters *k* and  λ by cross validation. **C.** MDS plot for LMC clustering with input data. **D.** Proportion matrix visualized as a barplot.

**Supplementary Table 5: Pearson correlations between estimateCellCounts proportions and MeDeCom LMC proportions.** *P* values corresponding to Pearson correlation coefficients >0.7 are bolded.

|  | | **CD8T** | **CD4T** | **NK** | **Bcell** | **Mono** | **Gran** |
| --- | --- | --- | --- | --- | --- | --- | --- |
| **LMC1** | r | 0.097 | 0.071 | -0.017 | 0.839 | 0.1 | -0.346 |
|  | *p* | 0.329 | 0.473 | 0.865 | **<0.0005** | 0.313 | <0.0005 |
|  | | | | | | | |
| **LMC2** | r | 0.733 | 0.142 | 0.669 | 0.082 | 0.075 | -0.537 |
|  | *p* | **<0.0005** | 0.15 | <0.0005 | 0.409 | 0.452 | <0.0005 |
|  | | | | | | | |
| **LMC3** | r | -0.051 | 0.702 | 0.077 | 0.014 | 0.122 | -0.425 |
|  | *p* | 0.606 | **<0.0005** | 0.438 | 0.887 | 0.219 | <0.0005 |
|  | | | | | | | |
| **LMC4** | r | 0.152 | 0.143 | 0.113 | 0.151 | 0.092 | -0.198 |
|  | *p* | 0.124 | 0.146 | 0.253 | 0.127 | 0.354 | 0.044 |
|  | | | | | | | |
| **LMC5** | r | -0.535 | -0.773 | -0.538 | -0.405 | -0.236 | 0.898 |
|  | *p* | <0.0005 | **<0.0005** | <0.0005 | <0.0005 | 0.016 | **<0.0005** |
|  | | | | | | | |
| **LMC6** | r | -0.054 | -0.048 | -0.022 | -0.115 | -0.068 | 0.093 |
|  | *p* | 0.588 | 0.625 | 0.826 | 0.244 | 0.491 | 0.346 |
